#  Influence of Peripheral Artery Disease and Statin Therapy on Apolipoprotein Profiles

**DOI:** 10.1155/2013/548764

**Published:** 2013-09-11

**Authors:** Andrew W. Gardner, Petar Alaupovic, Donald E. Parker, Polly S. Montgomery, Omar L. Esponda, Ana I. Casanegra

**Affiliations:** ^1^Reynolds Oklahoma Center on Aging, Donald W. Reynolds Department of Geriatric Medicine, University of Oklahoma Health Sciences Center (OUHSC), Oklahoma City, OK 73117, USA; ^2^Veterans Affairs Medical Center, Oklahoma City, OK 73104, USA; ^3^University of Oklahoma Health Sciences Center, O'Donoghue Research Building, 1122 NE 13th Street, Suite 1200, Oklahoma City, OK 73117, USA; ^4^Oklahoma Medical Research Foundation, Oklahoma City, OK 73104, USA; ^5^Department of Biostatistics and Epidemiology, OUHSC, Oklahoma City, OK 73117, USA; ^6^Cardiovascular Section, Department of Medicine, OUHSC, Oklahoma City, OK 73117, USA

## Abstract

Apolipoprotein B is a stronger predictor of myocardial infarction than LDL cholesterol, and it is inversely related to physical activity and modifiable with exercise training. As such, apolipoprotein measures may be of particular relevance for subjects with PAD and claudication. We compared plasma apolipoprotein profiles in 29 subjects with peripheral artery disease (PAD) and intermittent claudication and in 39 control subjects. Furthermore, we compared the plasma apolipoprotein profiles of subjects with PAD either treated (n = 17) or untreated (n = 12) with statin medications. For the apolipoprotein subparticle analyses, subjects with PAD had higher age-adjusted Lp-B:C (P < 0.05) and lower values of Lp-A-I:A-II (P < 0.05) than controls. The PAD group taking statins had lower age-adjusted values for apoB (P < 0.05), Lp-A-II:B:C:D:E (P < 0.05), Lp-B:E + Lp-B:C:E (P < 0.05), Lp-B:C (P < 0.05), and Lp-A-I (P < 0.05) than the untreated PAD group. Subjects with PAD have impaired apolipoprotein profiles than controls, characterized by Lp-B:C and Lp-A-I:A-II. Furthermore, subjects with PAD on statin medications have a more favorable risk profile, particularly noted in multiple apolipoprotein subparticles. The efficacy of statin therapy to improve cardiovascular risk appears more evident in the apolipoprotein sub-particle profile than in the more traditional lipid profile of subjects with PAD and claudication. This trial is registered with ClinicalTrials.gov NCT00618670.

## 1. Introduction

Peripheral artery disease (PAD) is a highly prevalent medical condition, [1] and it is associated with high prevalence of coexisting vascular diseases in the coronary, cerebral, and renal arteries [2, 3]. Consequently, PAD is a deadly [4, 5] and costly disease [6]. Many patients with PAD are physically limited by ambulatory leg pain [7, 8], resulting in baseline ambulatory and physical dysfunction [9, 10], low physical activity [11, 12], and poor health-related quality of life [13]. Although PAD is considered by many to be a benign disease, as 70 to 80% of patients have stable claudication that does not progress to worsening claudication or critical limb ischemia [2], PAD patients have increased rates of functional decline and mobility loss compared to those without PAD [14], leading to higher rates of hospitalization and loss of independence [15].

We have previously found that cardiovascular risk factors, such as dyslipidemia, are associated with impaired ambulation and vascular function in subjects with PAD and claudication [16, 17]. Dyslipidemia is typically evident by an elevation in low-density lipoprotein cholesterol (LDL-C). Recently, the multinational INTERHEART study showed that apolipoprotein B (apoB) was a stronger predictor of myocardial infarction than LDL-C [18], and it is inversely related to physical activity [19] and modifiable with exercise training [20]. Thus, apolipoprotein measures may be of particular relevance for subjects with PAD and claudication because dyslipidemia is prevalent in 85–90% of subjects [21], and physical activity levels are low [22]. 

The classification of plasma lipoproteins based on apolipoprotein composition instead of size and density provides a new way of characterizing plasma lipoproteins as these subclasses differ not only in their apolipoprotein composition but also in their metabolic properties [23, 24]. 

In subjects with severe PAD requiring revascularization, women with both localized aortic stenosis and diffuse segmental stenosis have impaired apolipoprotein profiles compared to controls [25], and men with aneurysmal and stenotic aortoiliac diseases have similar, abnormal profiles [26]. However, little is known about apolipoprotein measures in subjects with claudication. 

Statin therapy improves claudication measures [27], suggesting that lowering lipids has a role in improving symptoms. However, it is not clear whether apolipoproteins are improved with statin medications in subjects with claudication. Therefore the purposes of this study are (1) to compare plasma apolipoprotein profiles in subjects with peripheral artery disease (PAD) and claudication and in control subjects and (2) to compare the plasma apolipoprotein profiles of subjects with PAD either treated or untreated with statin medications. We hypothesized that subjects with PAD have impaired apolipoprotein profiles compared to controls and that those treated with statin medications have more favorable apolipoprotein profiles than untreated subjects.

## 2. Methods

### 2.1. Subjects

#### 2.1.1. IRB Approval and Informed Consent

The procedures used in this study were approved by the Institutional Review Board at the University of Oklahoma Health Sciences Center and by the Research and Development Committee at the Oklahoma City VA Medical Center. Written informed consent was obtained from each patient prior to investigation.

#### 2.1.2. Recruitment

Subjects participated in this study at the General Clinical Research Center, at the University of Oklahoma Health Sciences Center. Subjects with PAD and claudication were recruited by referrals from vascular and primary care clinics at the University of Oklahoma Health Sciences Center and the Oklahoma City VA Medical Center. Control subjects were recruited by newspaper advertisements for the assessment of cardiovascular risk factors in individuals without a history of cardiovascular diseases.

#### 2.1.3. Screening of the Intermittent Claudication Group

Subjects with claudication secondary to vascular insufficiency were included in this study if they met the following criteria: (a) a history of ambulatory leg pain [28] and (b) an ankle-brachial index (ABI) ≤ 0.90 [2]. Subjects were excluded for the following conditions: (a) absence of PAD (ABI > 0.90 at rest), (b) inability to obtain an ABI measure due to noncompressible vessels, (c) use of cilostazol and pentoxifylline initiated within three months prior to investigation, (d) active cancer, (e) end stage renal disease defined as stage 5 chronic kidney disease, and (f) abnormal liver function.

#### 2.1.4. Screening of the Control Group

Control subjects were included in this study if they met the following criteria: (a) no history of ambulatory leg pain [28] and (b) an ABI ≥ 1.00. Controls were excluded from this study for the following conditions: (a) an ABI < 1.00, (b) inability to obtain an ABI measure due to noncompressible vessels, (c) poorly controlled hypertension (resting systolic blood pressure > 200 mm Hg or resting diastolic blood pressure > 120 mm Hg), (d) history of cardiovascular disease, cerebrovascular disease, myocardial infarction, or peripheral revascularization, (f) active cancer, (g) end stage renal disease defined as stage 5 chronic kidney disease, and (h) abnormal liver function. 

### 2.2. Measures

#### 2.2.1. Plasma Apolipoproteins and Lipoprotein Lipids

Subjects arrived in the morning fasted but were permitted to take their usual morning medication regimen. Blood samples were drawn into chilled EDTA (1 mg/dL of blood) containing tubes after an overnight fast. 

 Blood was analyzed for fasting glucose concentrations as part of the automated chemistry battery (complete metabolic panel). Blood was also analyzed for fasting lipids, apoB, apolipoprotein C-III (apoC-III), and apolipoprotein subparticles consisting of Lp-B-II, Lp-A-II:B:C:D:E, Lp-B:E + Lp-B:C:E, Lp-B, Lp-B:C, Apo AI, Lp-A-I, and Lp-A-I:A-II. Total cholesterol, triglycerides, and high-density lipoprotein cholesterol (HDL-C) were measured by standardized enzymatic procedure [29, 30]. Very low-density lipoprotein cholesterol (VLDL-C) and low-density lipoprotein cholesterol (LDL-C) were estimated by Friedewald formula, and non-HDL-C was calculated as total cholesterol minus HDL-C. To put the apolipoprotein subparticles in better context, they can be compared to the density classes of lipoproteins. Densities of subparticles ranging solely in the atherogenic density classes of chylomicrons, VLDL-C, intermediate low-density lipoprotein cholesterol (ILDL-C), and LDL-C are Lp-B:E + Lp-B:C:E, Lp-B:C, and Lp-B-II. Densities of apolipoprotein subparticles in the density classes of VLDL, ILDL, LDL, and HDL_2_ are Lp-A-II:B:C:D:E and Lp-B. Densities of subparticles in the density classes of LDL-C, HDL_2_, HDL_3_, and very high-density lipoprotein cholesterol (VHDL-C) are Lp-A-I and Lp-A-I:A-II. Finally, the density of the only apolipoprotein subparticle that ranges solely within the HDL-C density classes of HDL_2_, HDL_3_, and VHDL is ApoAI.

 Blood was frozen at minus 70°C for subsequent analysis of apolipoprotein levels. ApoC-III was measured in the total plasma sample and in the heparin-manganese precipitate (HP), following reconstitution of the original volume. ApoC-III HP represents the apoC-III bound to apoB-containing lipoproteins. ApoC-III in the heparin-manganese supernate (HS) represents apoC-III bound to apoA-containing lipoproteins. The values for apoC-III HS were derived by subtracting apoC-III HP from total plasma apoC-III.

 Density-defined lipoprotein classes, including total cholesterol, triglycerides, and HDL-C, were measured as previously described [31, 32]. VLDL-C and LDL-Cl were measured according to the Friedewald formula [30]. ApoA-I, ApoB, and ApoC-III were determined by the immunoturbidimetric procedure of Riepponen et al. [33] using corresponding monospecific polyclonal antisera generated in this laboratory. ApoA-I:A-II lipoprotein subclasses were measured by the procedure of Marz et al. [34], ApoC-III was also measured in Apo-A-I-containing lipoproteins (ApoC-III HS) and in ApoB-containing lipoproteins (ApoC-III HP) [35]. ApoC-III bound to HS and HP was measured by electroimmunoassay [36]. The ratio of ApoC-III HS/ApoC-III HP is denoted as the ApoC-III ratio (ApoC-III R).

#### 2.2.2. Medical History and Physical Examination

After the blood samples were drawn, subjects were seen by a study physician. Demographic information, height, weight, cardiovascular risk factors, comorbid conditions, claudication history, ABI, and a list of current medications were obtained from a medical history and physical examination.

### 2.3. Statistical Analyses

Within each of the three groups, controls, PAD subjects treated with statin, and PAD subjects not treated with statin, measurement variables were summarized as means and standard deviations. Dichotomous variables were summarized as percent with attribute present. As preliminary analysis, the mean ages of the three groups were compared using a one-way ANOVA and sex distributions compared using Chi-Square test in Tables 2 and 3. Dichotomous variables in PAD groups were compared using single degree of freedom Chi-Square test. In order to adjust for the observed age difference, lipid measures were compared among the three groups using a one-way ANCOVA with age as covariate followed by two orthogonal contrasts—control mean minus average of two PAD group means and PAD no statin group minus PAD subjects with statin. Apolipoprotein means were compared in a similar manner. Computations were made using NCSS statistical package with significance defined as *P* < 0.05.

## 3. Results

### 3.1. Clinical Characteristics

 The clinical characteristics of the PAD groups and the control group are shown in Table 1. The groups were significantly different in age (*P* = 0.003), with the control group being the youngest. There was no group difference in the distribution of men and women (*P* = 0.695). Since the subjects in the healthy control group were required to be free of diabetes, hypertension, current smoking, and coronary artery disease, group comparisons on these variables were limited to the two PAD groups. In the PAD group taking statins, the percentage of subjects who had diabetes was higher than in the PAD group not taking statins (*P* = 0.005). The two PAD groups were not significantly different in the prevalence of hypertension (*P* = 0.144), current smoking (*P* = 0.176), and coronary artery disease (*P* = 0.913).

### 3.2. Lipid Measures

 Lipid measures of subjects with PAD and controls are displayed in Table 2. Triglycerides (*P* = 0.011), LDL cholesterol (*P* = 0.011), HDL cholesterol (*P* = 0.016), and the triglyceride/HDL cholesterol ratio (*P* = 0.005) were significantly different among the groups. The age-adjusted mean values of triglycerides (*P* < 0.01) and the triglyceride/HDL cholesterol ratio (*P* < 0.001) were significantly higher in the PAD groups than in the control group, whereas the HDL cholesterol (*P* < 0.05) and the LDL cholesterol (*P* < 0.01) were significantly lower in the PAD groups. The only age-adjusted mean that was significantly different between the two PAD groups was the LDL cholesterol/HDL cholesterol ratio, as the subjects on statin therapy had a lower value (*P* < 0.05).

### 3.3. Apolipoprotein Measures

 Apolipoprotein measures of subjects with PAD and controls are displayed in Table 3. Lp-B:C (*P* = 0.013) and Lp-A-I (*P* = 0.049) were significantly different among the groups. The age-adjusted mean value of Lp-B:C (*P* < 0.05) was significantly higher in the PAD groups than in the control group, whereas the Lp-B (*P* < 0.05) and Lp-A-I:A-II (*P* < 0.05) were significantly lower in the PAD groups. The age-adjusted mean values significantly different between the two PAD groups were ApoB (*P* < 0.05), Lp-A-II:B:C:D:E (*P* < 0.05), Lp-B:E + Lp-B:C:E (*P* < 0.05), Lp-B:C (*P* < 0.05), and Lp-A-I (*P* < 0.05), as the subjects on statin therapy had lower values.

## 4. Discussion

### 4.1. Subjects with PAD Compared to Controls

#### 4.1.1. Apolipoprotein Measures

To our knowledge, this is the first study to compare apolipoprotein subparticles in subjects with PAD and claudication compared to control subjects. The PAD groups had a 5 mg/dL higher age-adjusted Lp-B:C value than the control group, a 6 mg/dL lower age-adjusted Lp-B level, and a 9 mg/dL lower age-adjusted Lp-A-I:A-II value.

 The higher Lp-B:C particles level in the PAD group supports a previous study with type 2 diabetic patients that showed that levels of Lp-B:C particles were independently associated with macrovascular complications, as defined by the presence of coronary artery disease, PAD or cerebrovascular disease, or more than one of these [37]. The lower Lp-A-I:A-II particles level in the PAD group is supportive of other studies that found that these particles have some antiatherogenic potential, due to its role in reverse cholesterol transport as part of the HDL-C subpopulation. However, its true participation in the antiatherogenic or atherogenic process is still to be elucidated [24]. The impaired apolipoprotein subparticle measures of Lp-B:C and Lp-A-I:A-II in the subjects with PAD also support previous work that subjects with more severe PAD requiring revascularization had impaired apolipoprotein profiles compared to controls [25]. An unexpected finding was that the PAD group as a whole had lower Lp-B particles level than the control-group. This may be a reflection of the efficiency of the control-group in lipolytic degradation as Lp-B is the main lipoprotein degradation product [38].

#### 4.1.2. Lipid Measures

The PAD groups had a 60 mg/dL higher age-adjusted triglyceride level than the control group, a 2.3 higher age-adjusted ratio of triglyceride/HDL-C, an 11 mg/dL lower age-adjusted HDL-C concentration, and a 23 mg/dL lower age-adjusted LDL-C value. 

 The higher triglyceride level in the PAD group supports a previous observation of a 118% higher triglyceride concentration in subjects with diffuse, stenotic PAD compared to controls [25]. The impairments in triglycerides and HDL-C are both factors that cluster together and are components of metabolic syndrome [39]. We have previously found that metabolic syndrome is associated with worse claudication, physical function, health-related quality of life, and peripheral circulation in subjects with PAD [40] and that these factors are progressively impaired as the number of metabolic syndrome components increases. We have also found that dyslipidemia is associated with impaired calf muscle hemoglobin oxygen saturation during ambulation [16], which suggests that dyslipidemia impairs the microcirculation and may be a physiologic mechanism for worse ambulatory function. 

 Surprisingly, the PAD group had lower LDL-C than the controls, possibly because 17 of 29 subjects with PAD were taking statin medications. Since apoB was not different between the PAD and control groups, this suggests that subjects with PAD may have had higher values of intermediate-density lipoprotein cholesterol (IDL-C) and very low-density lipoprotein cholesterol (VLDL-C) because apoB directly measures the total number of atherogenic particles [41]. Thus, higher IDL-C and VLDL-C values would counteract lower LDL-C, resulting in similar apoB levels.

### 4.2. Statin Therapy in Subjects with PAD

 To our knowledge, this is the first study to examine the relationship between statin therapy and apolipoprotein subparticles in subjects with PAD and claudication. Subjects with PAD who were taking statin medications had lower values of LDL-C/HDL-C ratio, apoB, Lp-A-II:B:C:D:E, Lp-B:E + Lp-B:C:E, Lp-B:C, and Lp-A-I than subjects who were not taking statin medications. These findings agree with the observation that subjects with PAD who take statin medications have lower LDL-C [42] than those not on statins and that LDL-C is reduced with statin therapy [43]. Furthermore, our finding of a lower apoB level in subjects with PAD taking statin medications compared to those not taking statin medications suggests that they also had a lower number of atherogenic particles because there is only one apoB molecule on the surface of all LDL-C, IDL-C, and VLDL-C [41]. Our findings suggest that statin medications reduce the number of atherogenic particles in subjects with PAD, which may improve survival [42], event-free survival [42], and microcirculation during exercise [16].

 The effects of statins among apolipoproteins subparticles are not uniform and depend on both the type of pharmacological agent and the type of apolipoprotein subparticle being measured [24]. For example, although atorvastatin and simvastatin have similar profiles in the reduction of LDL-C, atorvastatin is better at reducing levels of Lp-B:C when compared to simvastatin [44]. However simvastatin is superior to atorvastatin in its capacity to lower levels of Lp-A-II:B:C:D:E [44]. In this study, we observed lower levels of apolipoprotein subparticles Lp-A-II:B:C:D:E, Lp-B:E + Lp-B:C:E, Lp-B:C, and Lp-A-I in the PAD statin group compared to the PAD no statin group. Given the LDL-C lowering effect of statin therapy, this is an expected pattern of results because these subparticles are direct constituents of LDL-C.

 Another observation was that Lp-A-I was lower in the PAD statin group. This was not expected because Lp-A-I is in the higher density gradient of apolipoprotein subparticles, and it is a major constituent of HDL-C. A possible explanation may be related to the polydisperse character of lipoprotein families within ApoA-I-containing lipoprotein subclasses, as many overlap with the LDL-C class. It is possible that the LDL-C lowering effect of statins resulted in concomitant reduction in the Lp-A-I subparticle.

 We have previously found that subjects with PAD receive suboptimal management for dyslipidemia, hypertension, and diabetes [45]. Based on our current observations, more optimal management of dyslipidemia in subjects with PAD may lower cardiovascular risk by favorably improving several apolipoprotein subparticles in addition to apoB.

### 4.3. Limitations

 There are limitations to this study. Subjects with PAD and controls who participated in this trial were volunteers and therefore may represent those who were more interested in exercise and in their health, who had better access to transportation to our research center, and who had relatively better health than PAD subjects and controls who did not volunteer. The cross-sectional design comparing those with and without PAD does not allow causality to be established, as it is possible that unfavorable apolipoprotein and lipid profiles may either precede or be a consequence of the development of PAD and claudication. The present findings are also limited to PAD subjects with a history of claudication. Thus, the current findings cannot be generalized to subjects with less severe PAD (i.e., asymptomatic PAD) or more severe symptoms (i.e., critical leg ischemia) or to those who are limited in their exercise performance by other significant comorbid conditions. Finally, this study consisted of small group sample sizes, and subjects were not recruited as a consecutive series.

## 5. Conclusions

Subjects with PAD have worse lipid profiles and impaired apolipoprotein profiles than controls, characterized by Lp-B:C and Lp-A-I:A-II. Furthermore, subjects with PAD on statin medications have a more favorable risk profile, particularly noted in multiple apolipoprotein subparticles. The efficacy of statin therapy to improve cardiovascular risk appears more evident in the apolipoprotein subparticle profile than in the more traditional lipid profile of subjects with PAD and claudication. The possibility of apolipoprotein subparticles as better therapeutic targets for the management of dyslipidemia is an attractive proposition as it may allow identifying patient populations that are at higher risk of developing adverse cardiovascular events and progression of PAD. Moreover the suggestion that statins may selectively alter levels of apolipoprotein subparticles is an endeavor worth pursuing but will need the rigors of a formal randomized control trial. Until then, these results are worth reporting as observed findings in the first study to compare apolipoprotein subparticles in subjects with PAD and claudication compared to control subjects. 

## Figures and Tables

**Table 1:** Clinical characteristics of subjects with peripheral artery disease (PAD) and controls. Values are means (standard deviation) or percentage of subjects in each category.

Variables	Control group (*n* = 39)	PAD no statin group (*n* = 12)	PAD statin group (*n* = 17)	*P* value
Age (years)	55 (13)	66 (13)	63 (11)	0.003
Sex (% men)	54	67	53	0.695
Diabetes (% yes)	—	0	47	0.005
Hypertension (% yes)	—	92	69	0.144
Current smoking (% yes)	—	67	41	0.176
Coronary artery disease (% yes)	—	33	35	0.913

**Table 2:** Lipid measures of subjects with peripheral artery disease (PAD) and controls. Values are means (standard deviation).

Variables	Control group (*n* = 39)	PAD no statin group (*n* = 12)	PAD statin group (*n* = 17)	ANCOVA *P* value	ΔAdjusted mean = control group − PAD groups	ΔAdjusted mean = PAD no statin group − PAD statin group
Triglycerides (mg/dL)	87.5 (41.9)	154.1 (116.9)	133.9 (92.3)	0.011	−60.6**	21.6
Total cholesterol (mg/dL)	181.5 (53.2)	177.8 (31.3)	157.1 (32.3)	0.062	23.4*	17.4
HDL-C (mg/dL)	52.4 (15.2)	44.3 (13.6)	46.8 (13.7)	0.016	10.7*	−3.9
LDL-C (mg/dL)	117.5 (38.6)	108.3 (28.0)	87.2 (26.6)	0.011	23.3**	20.2
LDL-C/HDL-C	2.35 (0.83)	2.65 (1.22)	1.92 (0.64)	0.078	−0.1	0.77*
Non-HDL-C (mg/dL)	129.1 (47.8)	133.5 (32.9)	110.3 (27.9)	0.158	12.6	21.3
Total cholesterol/HCL-C ratio	3.62 (1.10)	4.36 (1.60)	3.56 (1.04)	0.076	−0.5	0.86
Triglyceride/HDL-C ratio	1.93 (1.48)	4.19 (3.78)	3.50 (3.35)	0.005	−2.3***	0.82

*P < 0.05, **P < 0.01, and ***P < 0.001.

HDL-C: high-density lipoprotein cholesterol; LDL-C: low-density lipoprotein cholesterol.

**Table 3:** Apolipoprotein measures of subjects with peripheral artery disease (PAD) and controls. Values are means (standard deviation).

Variables	Control group (*n* = 39)	PAD no statin group (*n* = 12)	PAD statin group (*n* = 17)	ANCOVA *P* value	ΔAdjusted mean = control group − PAD groups	ΔAdjusted mean = PAD no statin group − PAD statin group
ApoB (mg/dL)	90.9 (12.3)	93.8 (16.1)	82.1 (19.2)	0.085	1.7	12.0*
ApoC-III (mg/dL)	10.1 (3.0)	10.1 (2.1)	10.0 (3.4)	0.890	0.4	0.0
ApoC-III HS (mg/dL)	6.6 (2.7)	6.9 (1.3)	6.5 (2.0)	0.507	0.6	0.2
ApoC-III HP (mg/dL)	3.4 (1.3)	3.1 (0.9)	3.5 (3.0)	0.823	−0.2	−0.3
ApoC-III ratio	2.26 (1.32)	2.37 (0.63)	2.64 (1.46)	0.552	0.19	−0.42
Lp-B-II (mg/dL)	90.4 (13.2)	83.6 (17.9)	81.5 (18.9)	0.213	6.9	2.4
Lp-A-II:B:C:D:E (mg/dL)	12.1 (5.8)	23.0 (33.4)	10.0 (4.7)	0.079047	−3.0	12.5*
Lp-B:E + Lp-B:C:E (mg/dL)	12.4 (4.5)	22.6 (26.4)	12.7 (5.6)	0.066	−4.2	9.5*
Lp-B (mg/dL)	59.8 (7.2)	50.6 (20.0)	54.8 (12.1)	0.127	6.1*	−3.8
Lp-B:C (mg/dL)	9.5 (4.0)	20.0 (21.1)	10.4 (4.8)	0.013	−5.1*	9.4*
Apo A1 (mg/dL)	132.5 (16.5)	134.8 (20.2)	124.7 (18.9)	0.156	5.4	9.2
Lp-A-I (mg/dL)	34.8 (4.4)	48.2 (37.9)	32.5 (6.0)	0.049	−3.6	15.1*
Lp-A-I:A-II (mg/dL)	97.7 (12.9)	87.0 (27.2)	92.1 (15.5)	0.134	8.8*	−5.3
B/A1 ratio	0.69 (0.14)	0.62 (0.13)	0.66 (0.17)	0.673	0.03	−0.03
Lp-B/C-III HP ratio	20.15 (8.46)	18.33 (8.88)	21.36 (9.34)	0.461	1.78	−3.54

*P < 0.05.
